# Longitudinal analysis of vaginal microbiota during IVF fresh embryo transfer and in early pregnancy

**DOI:** 10.1128/spectrum.01650-23

**Published:** 2023-10-26

**Authors:** Sofia Väinämö, Schahzad Saqib, Ilkka Kalliala, Kaisa Kervinen, Kaisu Luiro, Maarit Niinimäki, Mervi Halttunen-Nieminen, Seppo Virtanen, Pekka Nieminen, Anne Salonen, Tiina Holster

**Affiliations:** 1 Department of Obstetrics and Gynaecology, University of Helsinki and Helsinki University Hospital, Helsinki, Finland; 2 Human Microbiome Research Program, Faculty of Medicine, University of Helsinki, Helsinki, Finland; 3 Department of Metabolism, Digestion and Reproduction, Faculty of Medicine, Imperial College London, London, United Kingdom; 4 Department of Obstetrics and Gynaecology, Oulu University Hospital, Oulu, Finland; 5 Medical Research Center, Oulu University Hospital and University of Oulu, Oulu, Finland; Chengdu University, Chengdu, Sichuan, China

**Keywords:** infertility, *L. crispatus*, embryo transfer, vaginal microbiota, IVF

## Abstract

Non-*Lactobacillus*-dominated vaginal microbiota has been associated with poor gynecologic health and complications during pregnancy. Lactobacilli and especially *Lactobacillus crispatus* associate with good reproductive health and dominate the microbiota during healthy pregnancy. We examined whether the composition of vaginal microbiota at the time of fresh embryo transfer (ET) has an impact on the success of *in vitro* fertilization (IVF) and whether vaginal microbiota changes from IVF-ET to early pregnancy within individuals. Vaginal swab samples were collected from subfertile women at the time of IVF-ET (*n* = 76) and at the eighth gestational week (*n* = 21) from those who achieved clinical pregnancy. The microbiota composition was analyzed using 16S rRNA gene amplicon sequencing. *L. crispatus* was more abundant among the 30 women who achieved clinical pregnancy (46.9% vs. 19.1%, *q* = 0.039) and the 26 women who had live birth (43.3% vs. 23.1%, *q* = 0.32) compared to those who did not. Lactobacilli, mainly *L. crispatus* (76.2%), dominated all early pregnancy samples. Microbiota remained the same, i.e., *Lactobacillus*-dominated type in 52% (11/21), shifted from one *Lactobacillus-*dominated type to another in 24% (5/21), or shifted from mixed community to *Lactobacillus*-dominated type in 24% (5/21) women, but never from *Lactobacillus* dominance to non-lactobacilli dominance. Our results emphasize the role of *L. crispatus* in the success of IVF-ET and in early pregnancy. During pregnancy, the microbiota shifted toward *L. crispatus* dominance even if it was undetectable before pregnancy, indicating that most women hold a reservoir of this beneficial *Lactobacillus* in their reproductive tract.

**IMPORTANCE:**

Infertility is a global public health issue which leads many couples to seek fertility treatments, of which *in vitro* fertilization (IVF) is considered to be the most effective. Still, only about one-third of the women achieve live birth after the first IVF embryo transfer (IVF-ET). Factors affecting embryo implantation are poorly known, but the female reproductive tract microbiota may play a key role. Our study confirms the beneficial role of vaginal lactobacilli, especially *Lactobacillus crispatus,* in the probability of achieving clinical pregnancy and live birth following IVF-ET. Our findings regarding the intra-individual shift of vaginal microbiota between non-pregnancy and pregnancy states are novel and provide new information about the dynamics of microbiota in the early steps of human reproduction. These findings may help clinicians in their attempts to optimize the conditions for ET by microbiota screening or modulation and timing the ET when the microbiota is the most favorable.

## INTRODUCTION

Infertility is a global public health issue affecting up to 15% of couples at the childbearing age (1). This leads to many couples seeking infertility treatments of which *in vitro* fertilization (IVF) is considered to be the most effective (2). However, only a few IVF cycles lead to pregnancy and childbirth, since only about 30%–35% of women achieve live birth after IVF–embryo transfer (IVF-ET) (3, 4). For a successful IVF-ET, the fertilized egg or blastocyst must be able to attach and implant to a healthy endometrium. Along with the quality of the embryo (5) and the receptivity of the endometrium (6), the IVF-ET outcome may also be affected by the microbiota of the female reproductive tract (7).

Lactobacilli are known to be the most abundant bacteria in a healthy endometrial (8) and vaginal (9) microbiota, and the presence of other microbes than lactobacilli on the tip of ET catheters has been associated with poor outcomes of IVF (10, 11). Also, in vaginal smears examined by microscopy, the dominance of other bacteria than lactobacilli has been associated with a lower clinical pregnancy rate (12, 13) and live birth rate (14) following IVF treatment.

Studies using high-throughput DNA sequencing techniques have suggested that *Lactobacillus*-dominated endometrial (15, 16), cervical (17), and vaginal (7, 18) microbiota may have higher pregnancy rates after IVF-ET, compared to women with non-*Lactobacillus* dominance. In particular, *Lactobacillus crispatus* has been associated with a successful IVF-ET outcome (19, 20). In turn, vaginal dysbiosis, characterized by the dominance of other bacteria than lactobacilli, has been associated with recurrent implantation failure (21). *Gardnerella vaginalis, Fannyhessea vaginae,* and *Prevotella bivia* are bacteria typically associated with bacterial vaginosis (22, 23) and have also been associated with lower pregnancy rates in IVF (12), whereas low diversity of the vaginal microbiota, reflecting *Lactobacillus* dominance, has been implicated in higher clinical pregnancy rates in IVF (24). However, some studies have found opposite results regarding the effect of *Lactobacillus* dominance on IVF-ET results (25 – 27). The discrepant results may originate from relatively small sample sizes or differences in the clinical or other background characteristics of the study populations (24, 25).

The composition of microbiota is affected by pregnancy, mainly due to the increasing levels of circulating estrogen (28). During pregnancy, the composition of vaginal microbiota shifts toward *Lactobacillus* dominance (29) and changes postpartum, as serum estrogen levels fall dramatically (28, 30). In particular, *L. crispatus* has been the most abundant species in the vaginal microbiota in the first (31) and third (32) trimesters, whereas early miscarriage (33) and preterm birth (PTB) (34) have been associated with the non-*Lactobacillus*-dominated microbiota. The intra-individual transition in the composition of the vaginal microbiota during IVF-ET and between early pregnancy has not been explored before.

The aims of this study were to investigate whether the composition of the vaginal microbiota at the time of the fresh embryo transfer is associated with the probability of achieving a clinical pregnancy and live birth and how the vaginal microbiota composition changes between the time of the IVF-ET and early pregnancy.

## RESULTS

### Cohort characteristics

A total of 76 women underwent fresh IVF-ET cycles, of which 30 (39.5%) achieved clinical pregnancy, and two (2.6%) had biochemical pregnancy. None had an ectopic pregnancy. Four of the 30 (13.3%) women with clinical pregnancy ended up in a miscarriage, and altogether, 26 women (34.2%) had live births. Women who achieved clinical pregnancy were significantly younger than women who did not, as were the women with live births (Tables 1 and 2). Also, women with clinical pregnancy (86.7% vs 63.0%, *P* = 0.02) and live birth (92.3% vs 62.0%, *P* = 0.005) were more often nulliparous (no prior deliveries) compared with women with no clinical pregnancy or live birth. There were no significant differences between the women who achieved clinical pregnancy or live birth compared to those who did not in terms of body mass index (BMI), duration of infertility, smoking habits, and education. We also compared clinical factors that might have an impact on the success of implantation, including serum anti-Müllerian hormone (AMH) level, endometrium thickness, embryo stage, and other related clinical variables, between the pregnancy, and non-pregnancy groups and found no significant differences (Table 1).

**TABLE 1:** Background and clinical characteristics of the study population according to the achievement of clinical pregnancy

Variable	Pregnancy (*n* = 30)	Non-pregnancy (*n* = 46)	*P*-value
Age (years)			0.01
Mean (SD [range])	32.9 (4.3 [23–39])	35.1 (3.6 [24–40])	
BMI (kg/m^2^)			0.91
Mean (SD [range])	24.9 (3.8 [19.8–32.5])	24.8 (4.2 [17.4–36.0])	
Duration of infertility (months)*^a^*			0.49
Mean (SD [range])	40.1 (27.6 [12–156])	46.0 (30.2 [12–134])	
Cause of infertility, *n* (%)			
Endometriosis (*n* = 20)	10 (33.3)	10 (21.7)	
Male factor (*n* = 11)	2 (6.7)	9 (19.6)	
Tubal factor (*n* = 16)	5 (16.7)	11 (23.9)	
Anovulation (*n* = 6)	4 (13.3)	2 (4.3)	
Unexplained (*n* = 23)	9 (30.0)	14 (30.4)	
Type of infertility, *n* (%)			0.08
Primary (*n* = 44)	21 (70.0)	23 (50.0)	
Secondary (*n* = 32)	9 (30.0)	23 (50.0)	
Prior delivery, *n* (%)			0.02
Yes (*n* = 21)	4 (13.3)	17 (37.0)	
No (*n* = 55)	26 (86.7)	29 (63.0)	
Prior extrauterine pregnancy, *n* (%)^*a*^			1
Yes (*n* = 6)	2 (6.7)	4 (8.9)	
No (*n* = 69)	28 (93.3)	41 (91.1)	
Prior miscarriage, *n* (%)^*a*^			0.65
Yes (*n* = 17)	6 (20.0)	11 (24.4)	
No (*n* = 58)	24 (80.0)	34 (75.6)	
Smoking status, *n* (%)^*a*^			0.39
Current/former smoker (*n* = 33)	15 (50.0)	18 (40.0)	
Non-smoker (*n* = 42)	15 (50.0)	27 (60.0)	
Level of education, *n* (%)^*a*^			0.91
Low (comprehensive school, vocational secondary school) (*n* = 16)	6 (20.7)	10 (21.7)	
High (upper secondary school, university) (*n* = 59)	23 (79.3)	36 (78.3)	
AFC			0.22
Mean (SD [range])	17.7 (10.8 [1–49])	14.3 (6.7 [4–34])	
AMH (ng/mL)^*b*^			0.53
Mean (SD [range])	2.6 (1.9 [0.1–6.4)]	2.3 (1.6 [0.4–8.4])	
Stimulation protocol, *n* (%)			0.79
Agonist (*n* = 52)	20 (66.7)	32 (69.6)	
Antagonist (*n* = 24)	10 (33.3)	14 (30.4)	
Endometrium thickness (mm)			0.77
Mean (SD [range])	10.3 (2.7 [3.7–17.4])	10.2 (2.4 [3.3–15.6])	
The number of oocytes retrieved			0.59
Mean (SD [range])	9.9 (5.6 [3–22])	9.5 (6.5 [2–27])	
The number of mature oocytes^*b*^			0.74
Mean (SD [range])	8.2 (4.9 [3–19])	8.1 (5.5 [1–22])	
Fertilization, *n* (%)			0.12
Conventional IVF (*n* = 56)	25 (83.3)	31 (67.4)	
ICSI (*n* = 20)	5 (16.7)	15 (32.6)	
The number of normally fertilized eggs			0.3
Mean (SD [range])	5.8 (4.0 [1–15])	5.3 (4.5 [0–16])	
Age of embryo transfered (days)			0.82
Mean (SD [range])	3.1 (0.9 [2–5])	3.2 (1.0 [2–5])	
Stage of embryo			0.75
Cleavage stage (*n* = 62)	25 (83.3)	37 (80.4)	
Blastocyst (*n* = 14)	5 (16.7)	9 (19.6)	

^*a*^ Data missing in one case.

^*b*^ Data missing in five cases.

**TABLE 2:** Background characteristics of the study population according to the achievement of live birth

Variable	Live birth (*n* = 26)	No live birth (*n* = 50)	*P*-value
Age (years)			
Mean (SD [range])	32.5 (4.2 [23–38])	35.1 (3.6 [24–40])	0.005
BMI (kg/m^2^)			
Mean (SD [range])	25.2 (3.9 [19.8–32.5])	24.7 (4.1 [17.4–36.0])	0.54
Duration of infertility (months)^*a*^			
Mean (SD [range])	39.7 (28.3 [12–156])	45.7 (29.7 [12–134])	0.48
Cause of infertility, *n* (%)			
Endometriosis (*n* = 20)	9 (34.6)	11 (22.0)	
Male factor (*n* = 11)	2 (7.7)	9 (18.0)	
Tubal factor (*n* = 16)	5 (19.2)	11 (22.0)	
Anovulation (*n* = 6)	4 (15.4)	2 (4.0)	
Unexplained (*n* = 23)	6 (23.1)	17 (34.0)	
Type of infertility, *n* (%)			0.02
Primary (*n* = 44)	20 (76.9)	24 (48.0)	
Secondary (*n* = 32)	6 (23.1)	26 (52.0)	
Prior delivery, *n* (%)			0.005
Yes (*n* = 21)	2 (7.7)	19 (38.0)	
No (*n* = 55)	24 (92.3)	31 (62.0)	
Prior extrauterine pregnancy, *n* (%)^*a*^			1
Yes (*n* = 6)	2 (7.7)	4 (8.2)	
No (*n* = 69)	24 (92.3)	45 (91.8)	
Prior miscarriage, *n* (%)^*a*^			0.09
Yes (*n* = 17)	3 (11.5)	14 (28.6)	
No (*n* = 58)	23 (88.5)	35 (71.4)	
Smoking status, *n* (%)^*a*^			0.78
Current/former smoker (*n* = 33)	12 (7.7)	21 (42.9)	
Non-smoker (*n* = 42)	14 (53.8)	28 (57.1)	
Level of education, *n* (%)^*a*^			0.69
Low (comprehensive school, vocational secondary school) (*n* = 16)	6 (24.0)	10 (20.0)	
High (upper secondary school, university) (*n* = 59)	19 (76.0)	40 (80.0)	

^*a*^ Data missing in one case.

### Vaginal microbiota at the time of fresh IVF-ET

After taxonomic annotations and quality filtration (>500 reads, median of 27,794 reads), 75 samples were available for the bacterial analysis. One sample from a woman who achieved clinical pregnancy and live birth after IVF-ET was not available for analysis due to low read count.

First, associations between background and clinical variables and the vaginal microbiota were analyzed with permutational analysis of variance (PERMANOVA). The microbiota composition was associated with clinical pregnancy (*R* ^2^ = 0.07, *P =* 0.00007), woman’s age (*R* ^2^ = 0.05, *P* = 0.001), gravidity (*R* ^2^ = 0.09, *P* = 0.008), parity (*R* ^2^ = 0.04, *P* = 0.01), and history of preterm birth (*R* ^2^ = 0.03, *P* = 0.008). Based on these results as well as the clinical differences presented in Tables 1 and 2, age, parity, and gravidity were used to adjust the microbiota comparisons between the IVF success groups in order to study their independent association with microbiota composition. Other background variables, including prior urogenital infections, smoking, use of probiotics or antibiotics in the past 3 months, or the level of education, had no impact on the vaginal microbiota variation. Also, clinical variables such as AMH, infertility diagnosis, or factors related to the IVF treatment did not explain the microbiota variation (https://github.com/SchahzadSaqib/HEMI).

**Fig 1:**
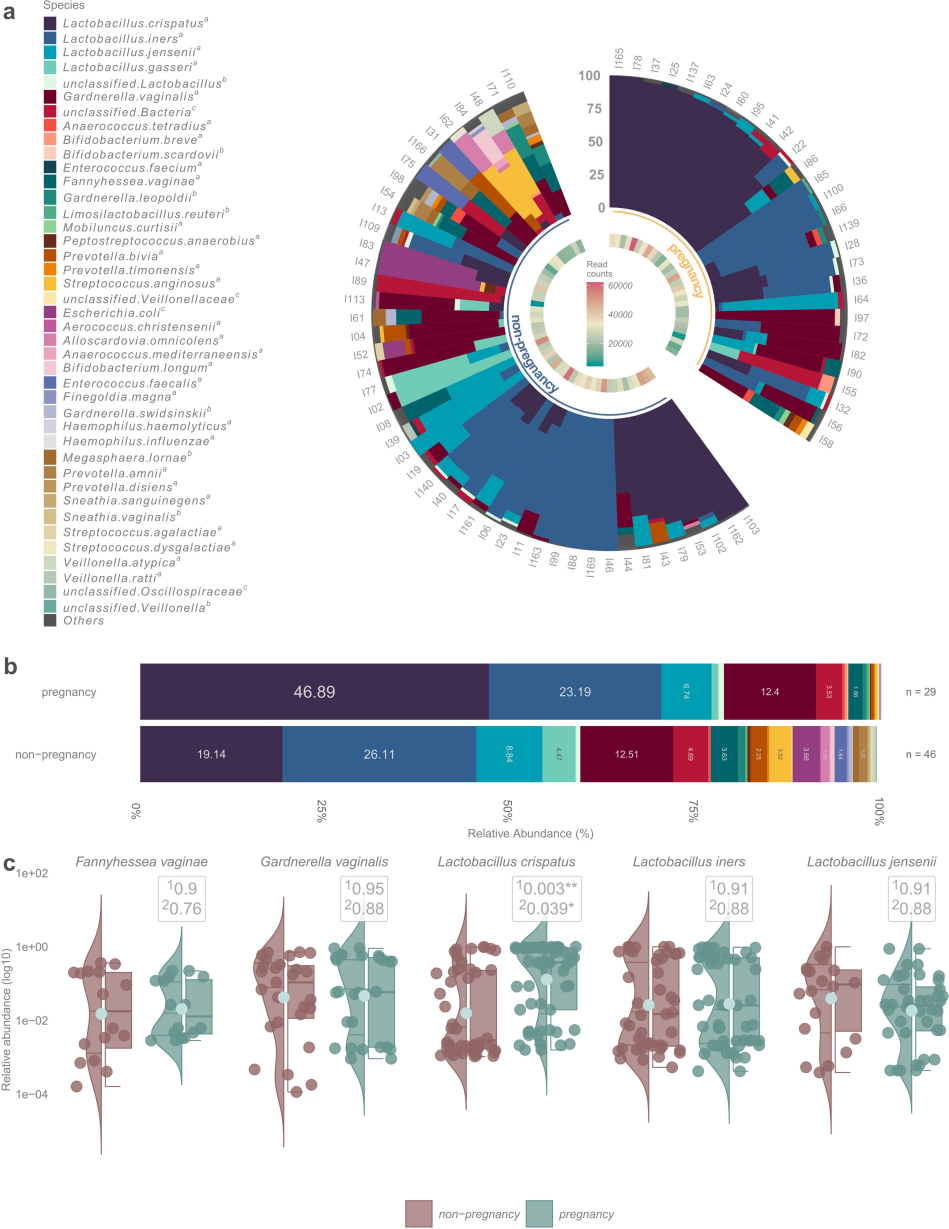
**(a**) Relative abundances of bacterial taxa in all 75 samples, divided based on pregnancy/non-pregnancy after fresh IVF-ET. (b**B** Stacked bar plot demonstrating the mean bacterial abundances of samples of women who achieved clinical pregnancy (*n* = 29) and women who did not (*n* = 46). **(c**) Violin plots showing the distribution of taxa between the groups. ^1^The non-adjusted value and ^2^after adjusting for age, parity, and gravidity.

**Fig 2:**
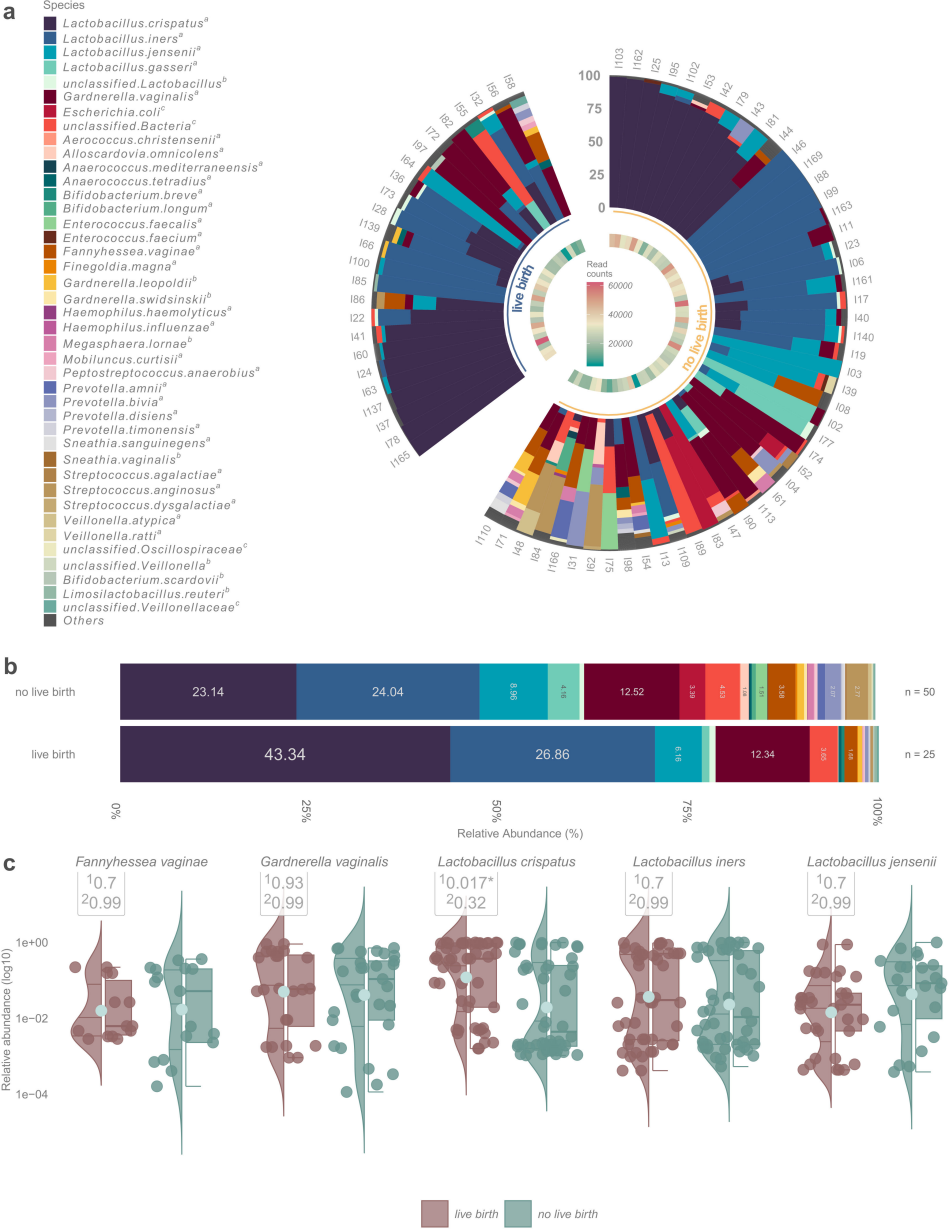
**(a**) Relative abundances of bacterial taxa in all 75 samples, divided based on the no live birth/live birth after fresh IVF-ET. **(b**) Stacked bar plot demonstrating the mean bacterial abundances of samples of women who achieved live birth (*n* = 25) and women who did not (*n* = 50). **(c**) Violin plots showing the distribution of taxa between the groups. ^1^The non-adjusted value and ^2^after adjusting for age, parity, and gravidity.

*Lactobacillus-*dominated (relative abundance >50%) vaginal microbiota was detected in 50 (66.7%) women at the time of IVF-ET (https://github.com/SchahzadSaqib/HEMI). Richness and diversity were on average lower for women who achieved clinical pregnancy, but the difference was not statistically significant (https://github.com/SchahzadSaqib/HEMI). *L. crispatus* was the most abundant species in the vaginal samples of women who achieved clinical pregnancy and live birth, whereas *Lactobacillus iners* was the most abundant species in those who failed to become pregnant (Fig. 1 and 2). Statistical analysis showed that the relative abundance of *L. crispatus* was significantly higher in the pregnancy vs non-pregnancy group (46.9% vs 19.1%, *q* = 0.003) and the result remained similar when adjusted for age, parity, and gravidity (Fig. 1). The relative abundance of *L. iners* (23.2% vs 26.1%, *q* = 0.91), *Lactobacillus jensenii* (6.7% vs 8.8%, *q* = 0.91), *G. vaginalis* (12.4% vs 12.5%, *q* = 0.95), and *F. vaginae* (1.9% vs 3.6%, *q* = 0.9) was slightly lower among women who achieved pregnancy compared to women who did not, but the differences were small.

In women who had live births, the relative abundance of *L. crispatus* was significantly higher compared to women with no pregnancy or live births (43.3% vs 23.1%, *q* = 0.017). However, when adjusted for age, parity, and gravidity the difference was not statistically significant (*q* = 0.32) (Fig. 2).

In the non-pregnancy group, *P. bivia* (8.70% vs 3.45%) and *Streptococcus anginosus* (6.52% vs 3.45%) were more prevalent and abundant (mean relative abundances 2.25% vs 0.39% for *P. bivia* and 3.02% vs 0.31% for *S. anginosus*) than in the pregnancy group, but the results were not tested for significance due to low overall prevalence in the cohort.

### Inter-individual comparison of microbiota at the time of the fresh IVF-ET embryo and at early pregnancy

**Fig 3:**
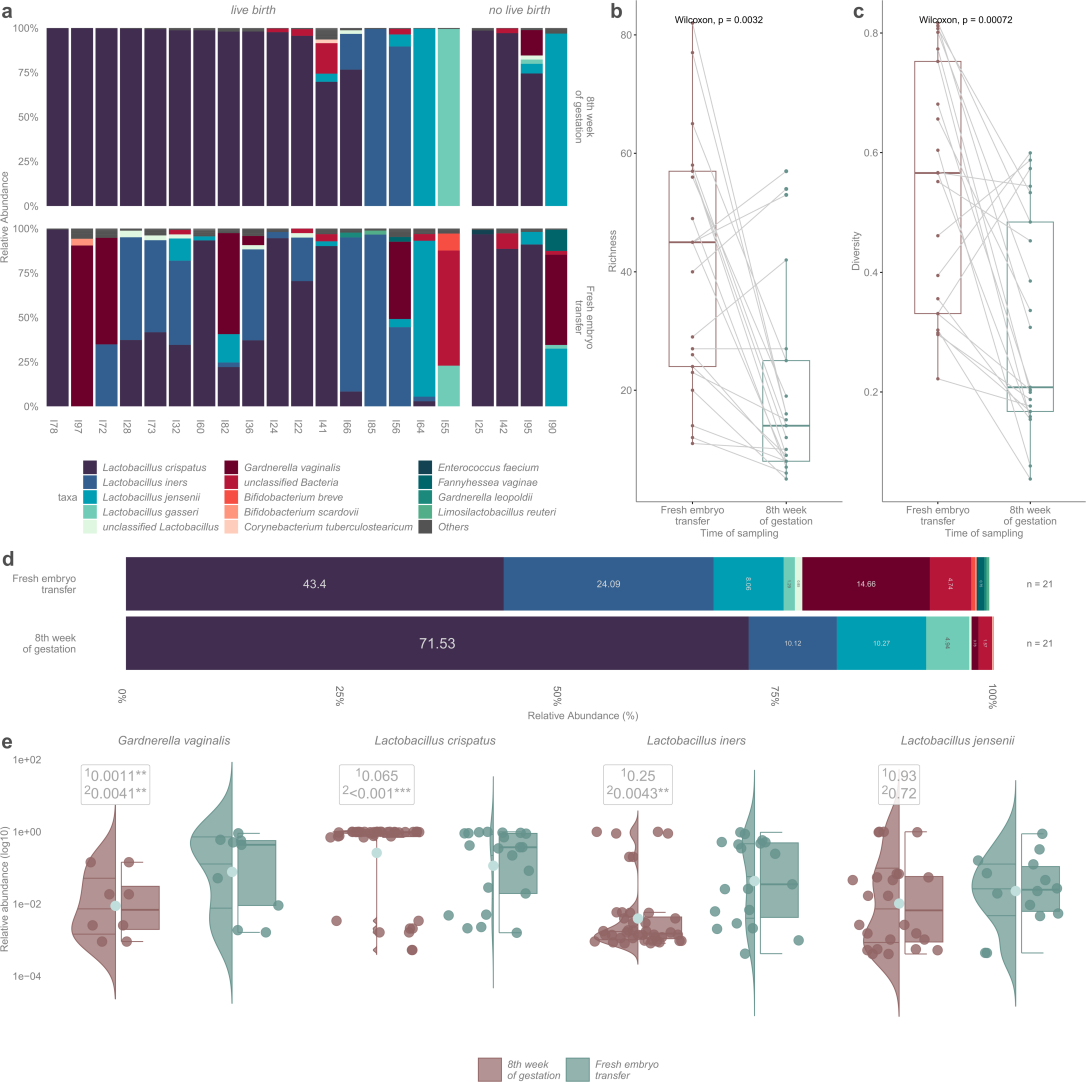
**(a**) Stacked bar plot demonstrating the inter-individual comparison of the microbiota of 21 women at the time of the ET and at early pregnancy. **(b**) Paired boxplot for richness comparisons between the longitudinal samples. (c) Paired boxplot for diversity comparisons between the longitudinal samples. (d) Stacked bar plot demonstrating the mean relative bacterial abundances between the samples taken from 21 women at the time of the fresh embryo transfer and at early pregnancy. **(e**) Violin plots showing the distribution of taxa between the different time points. ^1^The non-adjusted value and ^2^after adjusting for age, parity, and gravidity.

Vaginal samples were additionally collected at the eighth gestational week from 21 of the 30 women who achieved clinical pregnancy after ET. Nine samples were not available due to human error or because the ultrasound examination was performed elsewhere than in our research centers. Richness and diversity were significantly lower in samples at the eighth week of gestation (*P* = 0.0032; *P* = 0.00072, Fig. 3b and c). All pregnancy samples at 8 wk were dominated by lactobacilli, mainly by *L. crispatus* (*n* = 16, 76.2%). Two women (9.5%) had *L. iners*–dominated vaginal microbiota, two (9.5%) were dominated by *L. jensenii,* and one (4.8%) was dominated by *Lactobacillus gasseri* (Fig. 3). The relative abundance of *L. crispatus* was higher in pregnancy samples compared to the samples taken at ET (71.5% vs 43.4%, *q* = 0.065). The relative abundance of *L. iners* (24.1% vs 10.1%, *q* = 0.25) and *G. vaginalis* (14.7% vs 0.8%, *q* = 0.001) decreased between the IVF-ET and early pregnancy. After adjusting the results for age, parity, and gravidity, the relative abundance for *L. crispatus* (*q* < 0.001) and *L. iners* (*q* = 0.004) increased between the IVF-ET and early pregnancy (Fig. 3).

When comparing the vaginal samples taken at the time of the fresh IVF-ET and at early pregnancy in the same individuals, eight women had *L. crispatus* dominance in both samples, two women had *L. iners* dominance, and one woman had *L. jensenii* dominance in paired samples (Fig. 3). Altogether, 10 women showed a shift in their microbiota profiles between these two time points. In five women, microbiota dominance changed from *L. iners* to *L. crispatus*, in three women from *G. vaginalis* to *L. crispatus*, in one woman from *G. vaginalis* to *L. jensenii,* and in one woman from unclassified bacteria to *L. gasseri*.

## DISCUSSION

This study shows that the vaginal microbiota profile may influence the results of IVF-ET, as the dominance of *L. crispatus* at the time of the fresh IVF-ET was associated with a higher clinical pregnancy rate and higher live birth rate. In addition, a shift toward *Lactobacillus* dominance in early pregnancy was observed in women who successfully became pregnant after fresh IVF-ET.

Our results regarding the benefit of lactobacilli (35), especially on *L. crispatus,* and the success of implantation are in line with many earlier studies (7, 20). Koedooder et al*.* showed that women who had higher relative abundance of *L. crispatus* in their vaginal samples had higher chance of clinical pregnancy (7). Based on this finding, they developed an algorithm to predict the success of the first fresh IVF-ET based on the relative abundance of *L. crispatus* on the vaginal microbiota prior to the IVF cycle. However, they sampled women within 2 months before the analyzed IVF cycle, not at the time of IVF-ET. Another study consisting of women undergoing IVF with donated oocytes reported that women who achieved clinical pregnancy or live birth after fresh IVF-ET had higher abundance of *L. crispatus* compared to those who did not (19). There are also studies varying in sampling points during the IVF cycle and the type of embryo transferred (fresh or frozen) that have not found the composition of vaginal microbiota to affect the probability of achieving clinical pregnancy or live birth rate after IVF treatment (27, 36). Contradictory findings between different studies may also be explained by ethnically or geographically different study populations, since the vaginal microbial composition variates between different ethnic groups (9, 37).

The beneficial role of lactobacilli, especially *L. crispatus,* has been shown in several studies (38 – 40). *L. crispatus* possesses the enzymatic machinery to debranch and ferment the polymers derived from glycogen from the vaginal epithelium, producing lactic acid and hydrogen peroxide (H_2_O_2_), lowering the pH of the vagina (41, 42). Nevertheless, the function of hydrogen peroxide in the lower genital tract has still been controversial (43). Coupled with the secretion of bacteriocins, this acidic environment prevents the growth of other microbes and protects against sexually transmitted and other opportunistic infections (42, 44, 45). Lactic acid has also been shown to have immunomodulatory properties, particularly the suppression of inflammation and induction of anti-inflammatory compounds (42, 46, 47). Shortage of lactobacilli, in turn, has been associated with increased levels of proinflammatory cytokines (48). Hence, the vaginal microbiota overall can regulate the local immune environment and therefore contribute to the manifestation of various clinical phenotypes.

In the present study, *G. vaginalis*, *P. bivia,* and *S. anginosus* were more prevalent in the group that did not achieve pregnancy. This is in agreement with a previous study showing an increased relative abundance of *Streptococcus* and *Gardnerella* in women who failed to achieve a clinical pregnancy after fresh IVF-ET (49). Streptococci are often linked to aerobic vaginitis (AV) (50), a vaginal inflammation characterized by the disturbance of lactobacilli-dominated microbiota and increased amount of various aerobic bacteria (51), whereas *G. vaginalis, F. vaginae,* and *P. bivia* are known to be associated with bacterial vaginosis (BV) (22, 23). Both BV (12) and chronic endometritis (52) are suggested to decrease the likelihood of achieving clinical pregnancy in IVF-ET, whereas endometrial microbiota dominated by lactobacilli have been associated with higher implantation and ongoing pregnancy rates compared to non-lactobacilli-dominated microbiota (15). BV is known to compromise mucosal health in the vagina by the formation of high oxidative stress, degradation of mucin, and formation of polymicrobial biofilm initiated by *G. vaginalis* (53). While there are no studies on endometrial microbiota in BV, the intra-individual vaginal and endometrial bacterial communities have been observed to be closely related to each other (39, 54), and it is therefore likely that our results on the microbiota of vagina reflect the microbial environment of the uterine cavity as well. In parallel with the direct effects on the mucosa, non-*Lactobacillus-*dominated microbiota in the endometrium may trigger an inflammatory response that negatively affects embryo implantation and the likelihood of pregnancy, as immune mediators are tightly regulated during the implantation of the blastocyst to the endometrial epithelium (8, 15).

All vaginal samples taken at the eighth gestational week were dominated by lactobacilli in our study, even if the microbiota during the time of the IVF-ET in the same individual was non-*Lactobacillus* dominance. This result is in line with an earlier study, which showed that the relative abundance of *L. crispatus* in the vaginal samples of pregnant women is higher compared to those of non-pregnant women (29). However, this was a case–control study, and intra-individual changes in the microbiota composition between the non-pregnant and pregnant states were not studied. We showed that the relative abundances of *L. iners* and *G. vaginalis* were lower in early pregnancy samples than at the time of IVF-ET, indicating a shift in the composition of vaginal microbiota. In some women, *L. iners*–dominated microbiota shifted toward *L. crispatus* dominance between IVF-ET and early pregnancy, suggesting a shift toward a more stable microbiota type.

*L. iners* is a bacterium that can be dominant in both healthy women and in those with dysbiosis. By lacking the ability to produce D-lactic acid and hydrogen peroxide and by producing proteins mediating the adhesion of pathogens to the host cells, *L. iners* seems to provide less protection against the invasion of pathogens (55). Despite being a *Lactobacillus* species with beneficial characteristics such as the production of lactic acid, *L. iners*–dominated vaginal microbiota has been suggested to associate with BV, sexually transmitted infections, and even adverse pregnancy outcomes including PTB (55).

During a normal pregnancy, the vaginal microbiota profile is more stable than in the non-pregnant state (29). High levels of estrogen produced by the ovaries and the placenta promote glycogen stratification in the vaginal epithelial cells, which in turn induces the growth and proliferation of lactobacilli (56). In our study, bacterial communities remained in the same *Lactobacillus*-dominated type (52% of women), shifted from one *Lactobacillus-*dominated microbiota type to another (24%), or shifted from mixed community to *Lactobacillus*-dominant type (24%), but never from *Lactobacillus* dominance to non*-*lactobacilli-dominated microbiota type. This reflects the importance of lactobacilli in the vaginal ecosystem during early pregnancy protecting the uterus from ascending pathogens and the risk of miscarriage and PTB.

Our results regarding the intra-individual microbiota changes address a pivotal translational question of how to increase the carriage and abundance of lactobacilli that promote gynecological and reproductive health. In the current sample of 21 women, all possessed a *Lactobacillus*-dominant microbiota in favorable conditions, i.e., in the presence of high estrogen and resultant vaginal glycogen during pregnancy. Of these women, as high as 76% had *L. crispatus*–dominated microbiota during pregnancy, respectively. This indicates that most women carry *L. crispatus* in their vagina. Hence, approaches aiming to promote the existing strains deserve more attention as an efficient and safe option for microbiota restoration with, for example, prebiotics, over the current approaches on orally or vaginally supplemented probiotics, antibiotics, or vaginal microbiota transplants (57). Probiotic supplementation with *Lactobacillus* species has shown some benefit in maintaining healthy vaginal microbiota when used orally or vaginally (58, 59), although controversial results have been found as well (60). The only study that has examined the effect of probiotics in IVF detected no difference in the pregnancy rates between the vaginal probiotic and control groups (61). Similarly, the use of probiotics during the last 3 months did not have a significant effect on the outcome of the IVF treatment in our study.

Many studies suggest that the vaginal and endometrial ecosystems are not separate but share microbes (39, 54, 62). Thus, analyzing the microbial composition of vaginal microbiota at the time of IVF-ET could potentially be used as a screening test for the endometrium’s favorability for the implantation. We have shown before that the vaginal microbiota profile largely reflects endometrial microbiota profile intra-individually (39). Screening of vaginal microbiota at the time of IVF-ET would enable performing the ET in a cycle where the vaginal and accordingly the endometrial microbiota would be the most favorable for the implantation. However, further research is needed to evaluate how to promote the most favorable microbiota for implantation.

Strengths of this study include a prospective study design, uniform treatment and sample collection protocols in both participating tertiary centers, and state-of-the art methods for the analysis of the vaginal microbiota. The main limitation of our study was the small sample size and that only one IVF cycle was analyzed. Women had also different causes for their subfertility, and subjects with systemic diseases were not excluded, which may cause background variability. However, our study population was ethnically homogenous and composed of white women, which decreases inter-subject variation but may limit the generalizability of our results.

There is growing evidence that the vaginal microbiota may have an impact on reproductive health and the success of fertility treatments. Our results confirm the beneficial effect of *L. crispatus* on the outcome of the IVF treatment, particularly on the success of fresh embryo transfer. Our study is one of the few studies analyzing the outcome of fresh embryo transfer, and the samples were taken exactly at the time of the ET. To our knowledge, this is the first study to examine the intra-individual transition between the composition of vaginal microbiota during IVF-ET, i.e., before pregnancy and in early pregnancy.

In conclusion, vaginal microbiota and especially the presence of *L. crispatus* are positively associated with IVF-ET outcomes. Moreover, we report a shift of the vaginal microbiota toward *Lactobacillus* domination in early pregnancy also in women that had a non-*Lactobacillus*-dominated microbiota before pregnancy, suggesting that most women have an endogenous reservoir of lactobacilli with a potential to be promoted rather than supplemented externally. Our study provides novel information on the natural development of microbiota during early pregnancy and opens new research avenues regarding the optimization of the vaginal and endometrial microbiota.

## MATERIALS AND METHODS

### Study population

**Fig 4:**
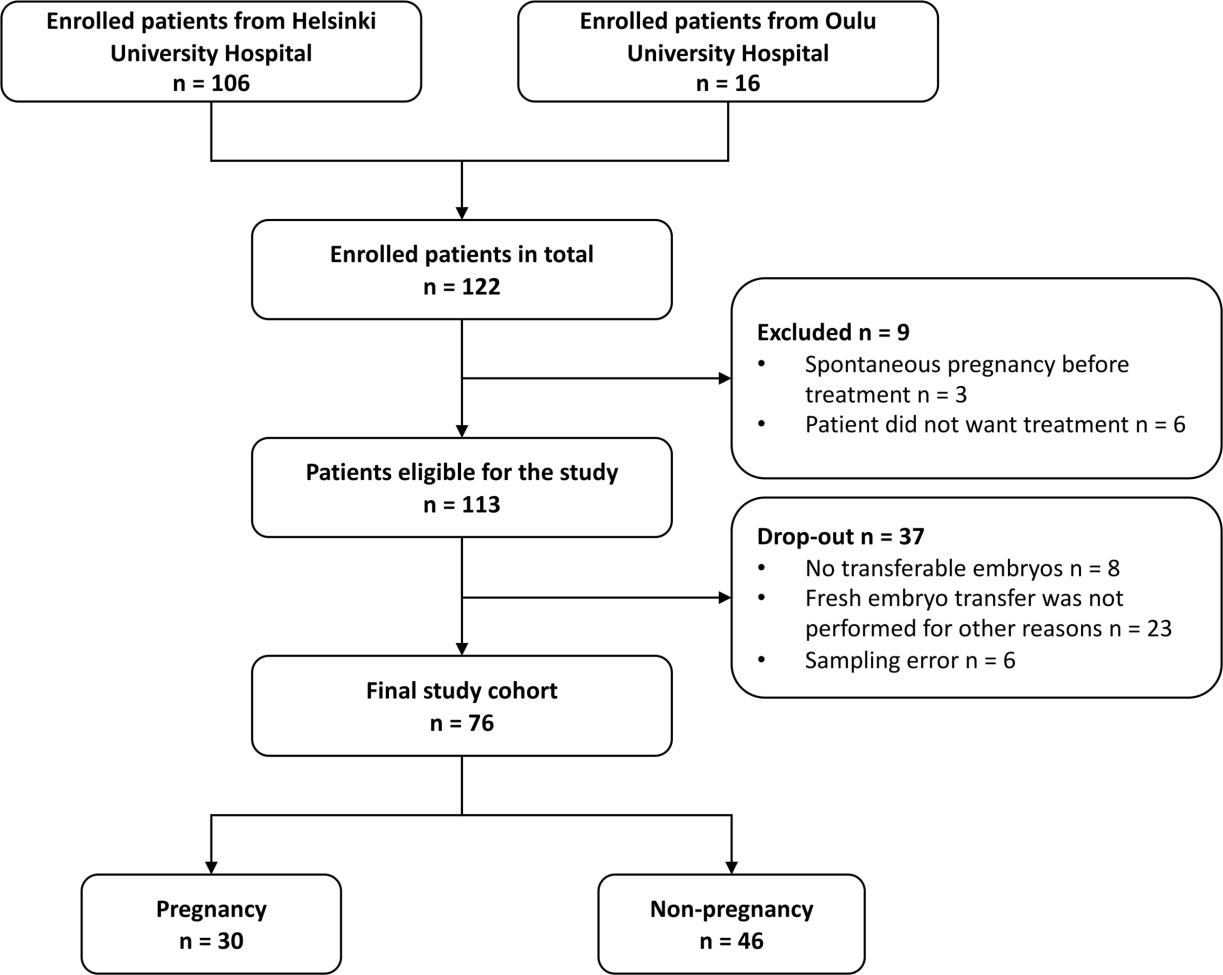
Flowchart of the study population.

This study was conducted in the Reproductive Medicine Unit, Helsinki University Hospital (Helsinki, Finland), and in Oulu University Hospital (Oulu, Finland). We recruited a total of 122 subfertile women undergoing one IVF treatment cycle with their own oocytes: 106 at Helsinki University Hospital between November 2019 and September 2021 and 16 at Oulu University Hospital between January 2020 and December 2020 (Fig. 4).

The patient recruitment was performed at the outpatient clinic visit, where the IVF treatment was planned 1–4 months prior to the treatment cycle. The inclusion criteria were women aged under 40 y with a male partner aged under 60 y, body mass index (BMI) under 35 kg/m^2^, and over 12 months of infertility. Six participants decided to forgo the planned treatment for personal reasons, and three had spontaneous pregnancy before IVF stimulation started. Of the remaining 113 women, 31 were excluded because there were no embryos after the stimulation cycle, or fresh embryo transfer was not performed for other reasons. In six women, samples were not collected due to human error. The reasons for withdrawal are summarized in https://github.com/SchahzadSaqib/HEMI.

The final study cohort was composed of 76 women between 23 yr and 40 yr of age who underwent one IVF stimulation cycle with either gonadotropin-releasing hormone (GnRH) agonist (*n* = 52) or antagonist (*n* = 24) protocol and underwent fresh embryo transfer. The main outcomes were clinical pregnancy rate, defined as the visualization of a gestational sac in an ultrasound at the eighth week of gestation, and live birth. Based on the outcome of the treatment, the population was divided into the non-pregnancy and pregnancy subgroups.

All women were asked to complete a background questionnaire about their gynecological history, sexual habits, previous infections, antibiotic and probiotic use, and educational status. Clinical data, including the outcome of the treatment, were obtained from the hospital’s patient registry.

### IVF treatment summary and sample collection

Women were treated by either GnRH agonist or antagonist stimulation protocol based on their individual medical backgrounds. In the agonist protocol, patient’s own GnRH production was suppressed using GnRH agonist nafarelin (Synarela) or leuprorelin (Procren) before ovarian stimulation. Once successful suppression was achieved, daily injections with follitropin alfa (Gonal-F) or menotropin (Menopur) were started to stimulate the development of the follicles. In the antagonist protocol, follitropin alfa injections were started from cycle day 2 or 3. In the midfollicular phase of the stimulation (5th–6th day of cycle), GnRH antagonist (ganirelix acetate) (Orgalutran or Fyremadel) was initiated to prevent spontaneous luteinizing hormone (LH) secretion and ovulation. Follicle development was monitored by transvaginal ultrasound examination. When the mean diameter of follicles was over 17 mm, with the presence of two or more follicles, ovulation was triggered by administering hCG injection (Ovitrelle or Pregnyl). Before oocyte retrieval, 13 (17.1%) women who had endometriomas received a single-dose cefuroxime 1.5 mg intravenously. Oocytes were retrieved 36 h after ovulation trigger using vaginal ultrasound-guided aspiration and fertilized by either conventional IVF or intracytoplasmic sperm injection (ICSI). ICSI was used for 20 couples when low sperm count was confirmed. Fresh embryo transfer was performed according to the recommended elective single-elective transfer protocol 2–5 d after oocyte retrieval, under transabdominal ultrasound guidance. Luteal phase support began 48 h after oocyte retrieval and continued for 12 d.

Vaginal swab samples were collected with sterile flocked swabs (FLOQSwabs, Copan spa, Italy) by healthcare professionals at the time of fresh embryo transfer and at the eighth gestational week from those women who got pregnant. Lubricants were not used during sampling. Samples were severed to 1.5  mL Eppendorf tubes which were frozen at −20°C immediately after sampling and further moved to −80°C within 4 wk.

### DNA extraction and sequencing

Using a bead-beating method, the bacterial DNA was extracted from the vaginal swabs and subjected to quality control checks, followed by amplification, index PCR, and Illumina MiSeq sequencing as described previously (63). The targeted V3–V4 region of the 16S rRNA gene was amplified using the following primers: 341F 5′-CCTACGGGNGGCWGCAG-3′ and 785Rev 5′-GACTACHVGGGTATCTAATCC-3′).

After initial quality control, the paired-end sequencing data were processed using the 16S rRNA gene workflow of the dada2 R package (v1.26) (64), obtaining amplicon sequence variants (ASVs). Next, species-level taxonomic annotations were assigned using NCBI BLAST and the nucleotide database as part of the taxminer R package workflow (65).

As a supplemental analysis based mainly on the dominance or absence of *Lactobacillus spp*. in the vaginal microbiota, the taxonomic profiles were sorted into community state types (CSTs) using the VALENCIA classification tool (66) (https://github.com/SchahzadSaqib/HEMI).

### Statistical analysis

Statistical analyses were performed using IBM SPSS Statistics, version 27 (IBM Corporation, Armonk, NY, USA) software for univariate data and R (v4.2.2, Rstudio). A two-sample *t*-test and Mann–Whitney *U* test were used to compare the continuous background variables between the pregnancy and non-pregnancy groups; Pearson d (χ^2^) and Fisher’s exact tests were used to compare the categorical variables. The relative abundance of individual bacterial taxa of the subgroups of clinical outcomes was tested for significance using the “GroupTest” function implementing generalized linear models using negative binomial distribution of the mare R package (67). “Presence/observation” of a bacterium was defined as >5%, and “dominance” was defined as >50% of the relative abundance in the sample. “Prevalence” was defined as the presence of the microbe above a threshold of 1% of the sample across all samples. All reported *P*-values from the “Grouptest” function were false discovery rate (FDR) adjusted for multivariable comparisons and reported as *q*-values. Richness (“specnumber” function), alpha diversity (“diversity” function, Simpson index), and permutational analysis of variance for associations between background variables and the microbiota (“adonis2” function) were obtained with the vegan R package (68). Richness and alpha diversity were compared between the groups using the Wilcoxon test.

## Data Availability

The sequencing data generated within this study has been deposited to the European Nucleotide Archive (ENA), project accession number PRJEB61794. All R scripts, supplemental material, and basic metadata used for data processing, statistical analysis, and visualization have been deposited to GitHub (https://github.com/SchahzadSaqib/HEMI) where they are publicly available. Additional information can be made available by the corresponding author upon reasonable request. For data protection reasons, full clinical data cannot be made public.
